# The “End Life” of the Grape Pomace Waste Become the New Beginning: The Development of a Virtuous Cycle for the Green Synthesis of Gold Nanoparticles and Removal of Emerging Contaminants from Water

**DOI:** 10.3390/antiox11050994

**Published:** 2022-05-19

**Authors:** Jennifer Gubitosa, Vito Rizzi, Anna Laurenzana, Francesca Scavone, Elena Frediani, Gabriella Fibbi, Fiorenza Fanelli, Teresa Sibillano, Cinzia Giannini, Paola Fini, Pinalysa Cosma

**Affiliations:** 1 Dipartimento di Chimica, Università degli Studi di Bari “Aldo Moro”, Via Orabona, 4-70126 Bari, Italy; jennifer.gubitosa@uniba.it; 2 Dipartimento di Scienze Biomediche Sperimentali e Cliniche “Mario Serio” Viale Morgagni, 50-50134 Florence, Italy; anna.laurenzana@unifi.it (A.L.); francesca.scavone@unifi.it (F.S.); elena.frediani@unifi.it (E.F.); gabriella.fibbi@unifi.it (G.F.); 3 Consiglio Nazionale delle Ricerche, Istituto di Nanotecnologia (CNR-NANOTEC) c/o Dipartimento di Chimica, Università degli Studi di Bari “Aldo Moro”, Via Orabona, 4-70126 Bari, Italy; fiorenza.fanelli@cnr.it; 4 Consiglio Nazionale delle Ricerche CNR-IC, UOS Bari, Via Amendola, 4-70126 Bari, Italy; teresa.sibillano@ic.cnr.it (T.S.); cinzia.giannini@ic.cnr.it (C.G.); 5 Consiglio Nazionale delle Ricerche CNR-IPCF, UOS Bari, Via Orabona, 4-70126 Bari, Italy; p.fini@ba.ipcf.cnr.it

**Keywords:** gold nanoparticles, grape pomace, Biomedicine, antioxidant, human dermal fibroblasts, ROS

## Abstract

During the last decades, the demand for processes developed according to the Circular Economy Principles has increased, searching for an alternative life for wastes. For this purpose, a one-pot green approach is exploited during this work to synthesize gold nanoparticles (AuNPs) by using grape pomace waste from *Vitis vinifera*. A raw aqueous extract of grape seeds, skin, and stems is used for AuNPs synthesis. UV-Vis, XPS, SEM, and ATR-FTIR spectroscopies demonstrate the main role of the extract’s polyphenolic components in stabilizing nanoparticles. XRD, DLS, and Zeta Potential analyses were used to characterize AuNPs. Moreover, the ionic strength, pH, and temperature role was investigated through the Surface Plasmon Resonance (SPR) band observation to assess AuNPs’ stability and photostability. For foreseeing the as-synthesized AuNPs’ potential use in cosmetic and biomedical fields as multifunctional platforms, their antioxidant, and skin-lightening properties were tested, together with their sunscreen ability. A preliminary in-vitro evaluation is reported about the AuNPs’ cytoprotective effects against H_2_O_2_ oxidative stress-induced in normal human dermal fibroblasts. Briefly, the possibility of reusing the grape pomace waste after the AuNPs synthesis as an adsorbent for the efficient removal of emergent contaminants is preliminarily discussed in the paper, further valorizing the use of waste according to a bio circular approach.

## 1. Introduction

Rather recently, the use of agriculture/food wastes has started to increase for obtaining green, sustainable, and renewable products, according to the principles of Circular Bioeconomy [1]. More specifically, with the aim to decrease the environmental impact [2], reducing the generation of waste through their recycling and reuse for converting them into value-added products is one of the main challenges to respect the Circular Economy and Bioeconomy principles [1,3,4]. Indeed, the Circular Economy introduces an optimization of the resources trying to decrease both resource input (material and energy) and output (product, by-product, and waste) per product/service offered. This could be possible by foreseeing a production system fed with products that are considered wastes or destined at low value-added targets [5,6].

In this context, by focusing attention on fruit wastes, which are mostly rich in bioactive substances of high value [2,6] and, among these, grape pomace (GP), derived from the wine production is very interesting. Indeed, the grape and the derived wastes are rich in bioactive molecules, and, not surprisingly, the reduced risk of chronic diseases ascribed to the grapes and wine consumption is well known. For example, in Italy, with particular reference to Sardinia, the daily and moderate consumption of red wine, rich in polyphenols, represents the secret of longevity [1]. Or there is the famous “French paradox”, which attributed red wine consumption as a way to justify the relatively low incidence of cardiovascular diseases among French people, despite their diet rich in saturated fats [7,8]. In 2018, the Food and Agriculture Organization (FAO-United Nations) estimated the grape production to be more than 79 million tons [9]. Accordingly, a huge quantity of derived solid wastes, such as GP, is generated, particularly after processes of pressing and fermentation [4,10]. For example, if 75% of produced grapes are used for wine production, about 20–30% is disposed of as waste by-products, the GP [4]. GP is a mixture of skins, pulp, seeds, and stalks. If, on the one hand, GP could be employed for wine alcohol production, and as fertilizer or animal feed, on the other hand, a large part of these wastes is disposed of as not useful waste. As a result, GP potentially can induce environmental concerns impacting nature and human life. Disposing of GP could have harmful effects on biodegradation due to the presence of polyphenols. Kalli et al. [4] reported that a high phenolic content could decrease the pomace’s pH, causing environmental problems. Therefore, the GP waste management can represent an important environmental issue [1,2,3,9], and its alternative and innovative reuse should be considered as a novel challenge, considering its content in chemicals (i.e., water, polysaccharides, insoluble proanthocyanidins, lignin, structural proteins, and phenols) interesting from an economic point of view. Therefore, although the pomace is considered low-value waste, the exploitation of the production, for example, of interesting nutraceutical products, should improve the well-being of human society, increasing the sustainability of the grape-wine chain production according to circular economy principles [1,2,3,4,10]. In the last years, particular attention has been devoted to the derived polyphenols [11,12,13,14,15,16,17]. For example, polyphenols can be used to synthesize nanomaterials by following a “green” and sustainable approach [11,12,13,14,15,16,17]. Many studies [11,12,13,14] in nanotechnology are inspired by nature to develop innovative methods. Indeed, green nanotechnology encompasses green chemistry and engineering principles intending to produce eco-friendly nanoparticles, avoiding the use of toxic chemicals, high temperature, high pressure, or energy [11]. For this purpose, several biodegradable food waste materials were employed in the past to synthesize metallic nanoparticles (NPs) [15]. The basic requirement of these materials is the content of different organic compounds such as polyphenols, flavonoids, carotenoids, and vitamins, which are involved in the synthesis and stabilization of NPs, acting as reductants and stabilizing agents in water [15]. In the case of GP wastes, the extracted polyphenols could act as reductant agents, specifically by reducing a solution of HAuCl_4_ from Au(III) to Au(0) by inducing the formation of nanoparticles [12,13,15,16]. So, starting from this background and intending to give GP an added value, this study aims to use GP, a mixture of seeds, skin, and stems, as a source of phenols to induce the formation of AuNPs by proposing eco-friendly multifunctional nanoplatforms. For this purpose, the raw material was simply washed with hot water, avoiding extraction techniques that could involve toxic chemicals, according to Green Chemistry principles. The obtained raw grape pomace wastewater extract (GWE) was used as obtained, without further purification, to reduce in a sustainable way Au(III), forming AuNPs. Although other studies are reported in the literature [10,11,16,17] about the use of GP as a source of phenols for the AuNPs synthesis, this work accounts for the first example of green synthesized AuNPs through the wastewater derived from GP. The Circular Bioeconomy is considered efficient not only if it exploits wastes searching high value-added products, but also if it makes a cascaded use of those [5]. For instance, as exhausted material after the AuNPs formation, the residual GP is proposed to be potentially employable as an adsorbent to remove emerging pollutants (EPs) from water, avoiding the disposal of a secondary by-product after the formation of AuNPs.

The problem related to EPs is another important issue in the last decades. For this purpose, several approaches have been investigated by the same authors of this paper to face the problem [18,19,20,21,22,23,24,25]. However, this work aims to present innovative waste management by extending its life, closing the gap present in literature about its use to remove EPs. Therefore, although the main paper focuses on the green synthesis of AuNPs, some information about the removal of EPs is presented and briefly discussed.

In particular, the obtained AuNPs were characterized from a physical and chemical point of view, using different techniques, such as UV-Vis and ATR-FTIR spectroscopies, TEM, XRD, and XPS analyses, in synergy. More specifically, the formation of AuNPs 30 nm wide, having a cubic Au phase, surrounded by an organic layer of phenols was demonstrated. Regarding the potential bio-application, first, the ABTS and the tyrosinase assays demonstrated the ability of AuNPs to act as antioxidants and tyrosinase inhibitors. The theoretical Sun Protection factor (SPF) was calculated to show that AuNPs can screen the UV radiation, potentially preventing sunburn and associated negative effects. More information was acquired by performing experiments in the presence of cells. Particularly, the cytoprotective effects of AuNPs against H_2_O_2_ oxidative stress-induced in normal human dermal fibroblasts were assessed. Finally, the used exhausted material, GP, after the extraction of active molecules for the AuNPs synthesis, is proposed to remove EPs from water, exploiting the dual life of this fruit waste. Ciprofloxacin (CIP) and Tetracycline (TC), highly dangerous antibiotics, were successfully removed from water using GP. It is worth mentioning that the removal of CIP should become a very actual and great problem. Recent studies have demonstrated that fluoroquinolone, and, thus, CIP, can be used in patients affected by pneumonia-induced COVID-19. Therefore, increased use of this drug is expected to face the ongoing pandemic situation, with the consequent increase in water pollution; as a result, novel, eco-friendly approaches must be searched, and GP can be further considered potential adsorbent material presenting its “end life” as a “new beginning”.

## 2. Materials and Methods

### 2.1. Chemicals

HAuCl_4_, ABTS, K_2_S_2_O_8_, tyrosinase (from mushroom lyophilized powder, ≥1000 unit/mg solid), tyrosine, NaCl, LiCl, KCl, MgCl_2_, CaCl_2_, NaBr, NaClO_4_, KH_2_PO_4_, KOH, HCl, and PerdrogenTM 30% H_2_O_2_ (*w*/*w*) were purchased from Sigma-Aldrich (Milan, Italy).

### 2.2. Grape Pomace Wastewater Extract Preparation

**Figure 1:**
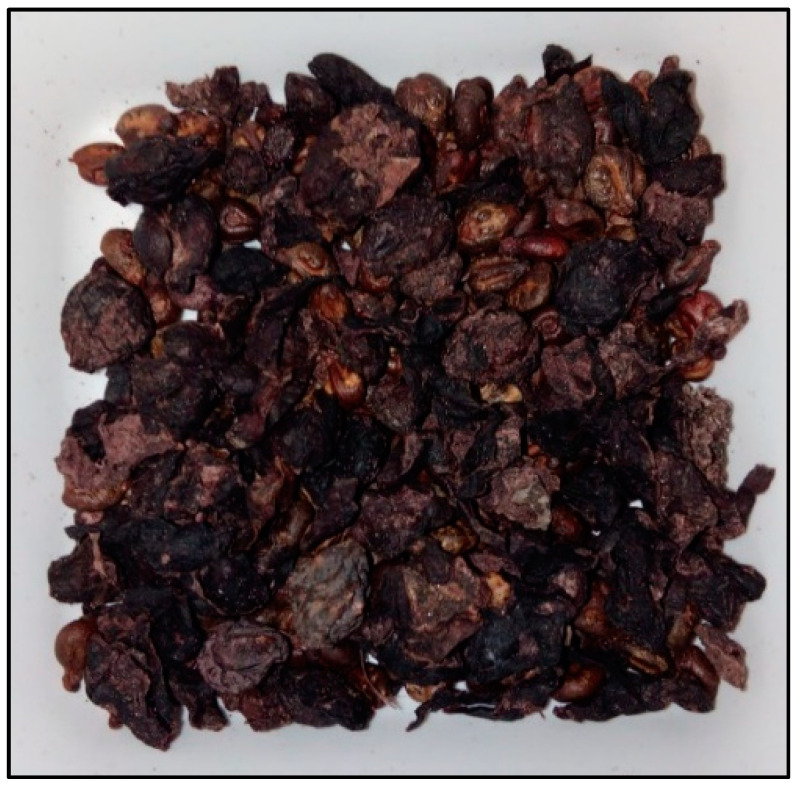
Camera picture of Grape Pomace Waste (seeds, skin, and stems).

Mixed grape waste (seeds, skin, and stems) (Figure 1) was procured from local wine producers and stored at −19 °C. Grape wastewater (GW) was obtained by adding 2 g of grape waste into 50 mL of deionized water, boiled for 5 min, and then separated from the solid residual by filtering the solution. The derived Grape Wastewater Extract (GWE) was thus centrifuged by using a Thermo Scientific Heraeus Multifuge X3R Centrifuge and stored at −19 °C before the use.

### 2.3. AuNPs Synthesis

A stock solution of HAuCl_4_ with a concentration of 1 × 10^−3^ M was prepared in deionized water. An appropriate amount of this solution was mixed with 1.750 mL of GWE to obtain an HAuCl_4_ concentration of 1.25 × 10^−4^ M. A final volume of 2 mL was used. The resulting solution was then moderately stirred. Different contact times as 1, 2, 4, 6, and 24 h were adopted to find the best experimental condition to obtain the AuNPs sample. The samples were centrifuged at 8000 rpm for 20 min using a D2012 High Speed Mini Centrifuge and washed with fresh deionized water to remove unreacted Au (III) and GWE. Considering the AuNPs’ average size derived from TEM analysis, a molar absorption coefficient of 3.36 × 10^9^ M^−1^cm^−1^ was adopted to infer their concentration. Therefore, using the UV-Visible spectroscopy and the Lambert–Beer law, a mean AuNPs concentration of 1 × 10^−11^ M from each synthesis was calculated, corresponding to 1 mg/mL of AuNPs. Particularly, considering the used molar absorption coefficient and the AuNPs size, the stock solution contained 1.79 × 10^11^ nanoparticles/mL. Before collecting UV-Visible spectra, the samples were diluted 1:5 with water.

### 2.4. UV-Visible Measurements

The UV-Visible absorption spectra of AuNPs solutions were collected using a Varian CARY 5 UV-Vis-NIR spectrophotometer (Varian Inc., now Agilent Technologies Inc., Santa Clara, CA, USA) in the range of 200–800 nm at a 1 nm/s scan rate. Measurements were performed using a cuvette with a 1 cm path length.

### 2.5. ATR-FTIR Spectroscopic Measurements

ATR-FTIR spectra were recorded within the 400–4000 cm^−1^ range using a Fourier Transform Infrared spectrometer (FTIR Spectrum Two from Perkin Elmer, Waltham, MA, USA) resolution of 4 cm^−1^. Sixteen scans were summed for each acquisition.

### 2.6. Transmission Electron Microscopy (TEM)

The analysis was performed by a JEOL JEM-1011 microscope operating at 100 kV. The TEM samples were prepared by casting a drop of AuNPs aqueous solution onto a carbon-coated copper TEM grid (400 mesh) and letting the solvent dry at room temperature.

### 2.7. X-ray Photoelectron Spectroscopy (XPS)

XPS measures were carried out employing a PHI P5000 VersaProbe II scanning XPS microprobe spectrometer (Physical Electronics GmbH, Feldkirchen, Germany), using a monochromatic Al Kα X-ray source (1486.6 eV) operated at a power of 24.8 W, with a spot size of 100 μm. Wide scans (0–1400 eV) and high-resolution spectra (C 1s, O 1s, N 1s, Au 4f, Na 1s, K 2p) were acquired in fixed analyzer transmission (FAT) mode with a pass energy of 117.40 and 23.50 eV, respectively. Spectra were collected at an angle of 45° with respect to the sample normal. Surface charging was compensated using a dual-beam charge neutralization system. The binding energy (BE) scale was corrected taking as reference the hydrocarbon component of the C 1s spectrum at 284.8 ± 0.2 eV. XPS analyses were repeated on three different spots of a sample prepared by drop-casting an aqueous nanoparticle dispersion onto a silicon substrate. The data processing was performed by using MultiPak software (Version 9.5.0.8, 30 October 2013, Ulvac-PHI, Inc., Lake Drive East, Chanhassen, MN, USA). After Shirley background subtraction, the high-resolution XPS spectra were fitted with mixed Gaussian–Lorentzian peaks. The uncertainty associated with peak position was estimated to be ±0.2 eV. A maximum relative standard deviation of 10% was estimated on the surface atomic concentrations and on the area percentages of the curve-fitting components.

### 2.8. X-ray Powder Diffraction (XRD)

XRD data were collected at room temperature from a sample of AuNPs by using a Bruker D8 Discover diffractometer (operating conditions 40 kV, 40 mA) equipped with a Goebel mirror for copper radiation (λKα1 = 1.54056 Å, λKα2 = 1.54439 Å) and a scintillator detector. A drop of colloidal solutions was deposited on a silicon zero-background substrate. Diffraction data were collected in a reflection geometry mode at a fixed incidence angle of ω = 1° while moving the detector between 20° and 100°, with a step size of 0.05°. The qualitative analysis of the crystalline phase content was performed using the QUALX 2.0 program [26].

### 2.9. Zeta Potential and Size Measurements

The average of the hydrodynamic diameters of AuNPs was measured by dynamic light scattering and using a Zetasizer Nano ZS (Malvern Instruments Ltd., Worcestershire, UK). In addition, the surface charge of AuNPs was measured as Zeta potential by laser doppler velocimetry using the same instrument.

### 2.10. AuNPs Photostability

The AuNPs photostability was assessed by irradiation with a solar simulator lamp purchased from Oriel Corporation, Stratford, CT, USA, Model 6684. A Xenon lamp (150 W) with an E0: 1482 mW/cm^2^~1.48 suns was used. A 1 cm quartz cuvette containing the sample, placed at 6.5 cm from the source, was used for the purpose. The AuNPs aqueous solution was exposed for different time intervals, and the UV-Vis absorption spectra were collected.

### 2.11. AuNPs Thermo-Stability

A heating magnetic stirrer (Arex, Velp Scientifica, Usmate Velate, Italy) controlled with an MGW Lauda R42/2 digital thermometer was used for performing thermostability experiments. Experimentally, the AuNPs aqueous solution was heated at different temperature values, and the UV-Vis absorption spectra were collected.

### 2.12. Determination of the Theoretical Sun Protection Factor

As already reported by Gubitosa et al. [12], an empirical mathematical expression (Equation (1)) for calculating the theoretical SPF in the UVB region was used, and, for this purpose, UV spectrophotometry was employed.

(1) $$SPF_{Spectrophotometric}=CF\times \sum_{290}^{320}EE\left(\lambda \right)\times I\left(\lambda \right)\times Abs\left(\lambda \right)$$

where *EE(λ*) is the erythemal effect spectrum; *I*(*λ*)*,* the solar intensity spectrum; *Abs*(*λ*) is the absorbance of standard solutions of the sunscreen product in the wavelength region 290–320 nm; and *CF*, the correction factor (=10). The values of *EE*(*λ*) × *I*(*λ*) are constant values obtained from the literature.

### 2.13. ABTS Assay

The ABTS was solubilized in water to obtain a 7 mM concentration. ABTS radical cation (ABTS^•+^) is produced from the reaction of the ABTS stock solution with 500 µL of potassium persulfate (0.6 mg/mL). The mixture, before the use, was left for 12 h in the dark at room temperature. Subsequently, the solution was diluted at 1:10 in water. By starting from this mixture, the inhibition studies were performed by diluting the latter solution (1:6) in water in the presence of appropriate amounts of the samples object of study. Equation (2) was used to calculate the total antioxidant activity:

(2) $$\%\;of\;antioxidant\;activity=\frac{A_{ABTS}-A_{Sample}}{A_{ABTS}}\times 100$$

where, $A_{sample}$ is the absorbance value reads at 800 nm of the solution containing AuNPs and ABTS after 1 h of incubation time, while $A_{ABTS}$ is the absorbance value of the ABTS solution read at the same wavelength before the addition of AuNPs. Before collecting the UV-Visible spectra, the samples were centrifugated to eliminate the contribution of the SPR band related to AuNPs.

### 2.14. Tyrosinase Assay

A tyrosinase stock solution 1000 U/mL in phosphate buffer 5.0 × 10^−2^ M, pH 7.5, and properly diluted at 1:600 was used. A tyrosine solution 2 mM was prepared by adding HCl to dissolve the amino acid in the buffer. The assay was performed in the dark by adopting 4 mL as the final volume. A volume of 2 mL of the diluted tyrosinase solution was reacted with 160 µL of tyrosine and 1 mL of the AuNPs sample. After adding tyrosine to the tyrosinase solution, the reaction was immediately followed by measuring the absorbance at 475 nm, indicative of the dopachrome formation. The effect of the contact time was also investigated. Equation (3) was used to calculate the % of dopachrome inhibition:

(3) $$\%\;\mathrm{of}\;\mathrm{inhibition}=\frac{A_{475\;\left(B\right)}-A_{475\;\left(S\right)}}{A_{475\left(B\right)}}\times 100$$

where A_475(S)_ is the absorbance value at 475 nm of solutions containing AuNPs and tyrosine/tyrosinase at different contact times, and A_475(B)_ is the absorbance value of solutions in the absence of AuNPs. Before each analysis, the samples containing AuNPs were centrifuged to remove the contribution of AuNPs in the spectra.

### 2.15. Cell Culture and Treatment

Normal Human Dermal Fibroblasts (NHDFs) (Lonza, Euroclone, Pero, Italy) were cultured in Dulbecco’s modified Eagle’s medium (Thermo Fisher Scientific, Inc., Waltham, MA, USA) supplemented with 10% fetal bovine serum (Sigma-Aldrich; Merck KGaA, Darmstadt, Germany) at 37 °C in an atmosphere containing 5% CO_2_. The experiments were conducted starting from pre-senescent fibroblasts (PDL = 24), where PDL stands for population doubling level, and it is calculated according to the equation: $PDL=3.32\times \log N/N_{0}$ (where *N* and *N*_0_ are the recovered and seeded cell numbers, respectively). Senescence was assessed based on beta-galactosidase staining [27]. H_2_O_2_ (Sigma-Aldrich; Merck KGaA) was diluted in phosphate-buffered saline (PBS) to obtain a 1 M stock solution. For the H_2_O_2_ treatment experiments, the H_2_O_2_ stock solution was diluted in cell culture media at the indicated concentration.

### 2.16. Cell Viability Assay

The viability of NHDFs was determined by trypan blue staining. Cells (1.0 × 10^5^) were seeded in a growth medium in 60-mm culture dishes and allowed to attach overnight. The next day, AuNPs colloidal solution was added at concentration 5 × 10^−13^ M for 15 h before H_2_O_2_ treatment. Fifteen hours later, 500 µM of H_2_O_2_ was added and incubated at 37 °C for 2 h. Subsequently, 20 μL of cell suspension were aseptically transferred to a 1.5 mL clear Eppendorf tube and incubated for 3 min at room temperature with an equal volume of 0.4% (*w*/*v*) trypan blue solution prepared in 0.81% NaCl and 0.06% (*w*/*v*) dibasic potassium phosphate. Viable and non-viable cells (trypan blue positive) were counted separately using a dual-chamber hemocytometer and a light microscope. The means of three independent cell counts were pooled for analysis.

### 2.17. SA-β-Galactosidase Assay

A-β-Galactosidase Assay SA-β-Gal staining was carried out by means of the Senescence b-Galactosidase Staining Kit (Cell Signaling Technology, Danvers, MA, USA). Staining was evident in 2–4 h and maximal in 12–16 h. The next day hematoxylin was used to counter-stain the cells.

### 2.18. Intracellular ROS Scavenging Assay

Intracellular Reactive Oxygen Species (ROS) production was evaluated using a 2′7′-dichlorofluorescein diacetate (DCF-DA; Sigma-Aldrich; Merck KGaA) staining assay. Briefly, cells were incubated with DCF-DA solution (100 µM) at 37 °C for 30 min after AuNPs and H_2_O_2_ treatment. ROS levels were then analyzed using flow cytometry. The proportion of fluorescence-positive cells was measured using a FACSCalibur flow cytometer (BD Biosciences, San Diego, CA, USA) with excitation and emission filters of 488 and 530 nm, respectively [28].

### 2.19. Western Blot Analysis

Harvested cells were resuspended in 20 mM RIPA buffer (pH 7.4) (Merk Millipore, Vimodrone, MI, Italy) containing a cocktail of proteinase inhibitors (Calbiochem, Merck, Darmstadt, Germany) and treated by sonication (Microson XL-2000, Minisonix, Farmingdale, NY, USA). Aliquots of supernatants containing equal amounts of protein (30 µg) in Laemmli buffer were separated on Bolt^®^ Bis-Tris Plus 4–12% precast polyacrylamide gels (Life Technologies, Monza, Italy). Fractionated proteins were transferred from the gel to a PVDF nitrocellulose membrane using an iBlot 2 system (Life Technologies, Monza, Italy). Blots were stained with Ponceau red to ensure equal loading and complete transfer of proteins and then blocked for 1 h at room temperature with 5% milk in PBS containing 0.1% Tween. Subsequently, membranes were probed at 4 °C overnight with rabbit anti-γH2AX antibody or Phospho-Stat5 (Tyr694) (Cell Signaling, Danvers, MA, USA) or anti-GAPDH antibody (1:1000 Cell Signaling) used to assess equal amounts of protein loaded in each lane. Anti-Rabbit IgG (whole molecule)–Peroxidase antibody (Sigma, St. Louis, MI, USA) were used as secondary antibodies; the ECL procedure was employed for development.

## 3. Results and Discussion

**Scheme 1:**
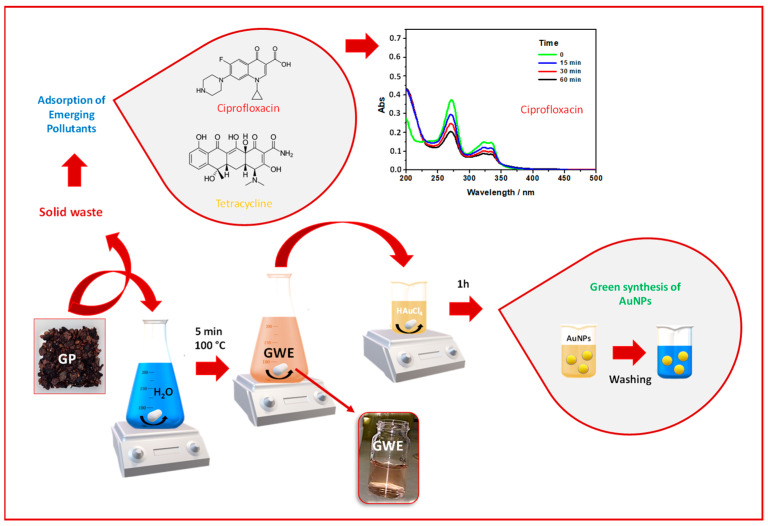
The scheme depicts the aim of the present work. First, GP is placed in boiled water to obtain the raw GWE needed to induce the AuNPs formation from HAuCl_4_. The exhausted GP, after the extraction, is reused to remove Ciprofloxacin (see the UV-Vis spectra collected during the adsorption process) and Tetracycline from water.

Based on Green Chemistry and Circular Economy principles, the proposed process aims to present Grape Pomace as a resource. The approach was performed by following two steps: first, the GP was placed in boiling water to remove dirt and obtaining raw GWE; subsequently, the GWE was used for synthesizing AuNPs, while the exhausted GP was employed as adsorbent material to remove two EPs (i.e., CIP and TC), from water, as reported in Scheme 1. It is worth specifying that the main topic of the present work is the AuNPs’ synthesis and their application in the biomedical field. In contrast, the environmental application of the exhausted grape pomace is only preliminary proof of opening new challenges in this field.

### 3.1. Green Synthesis of AuNPs

**Figure 2:**
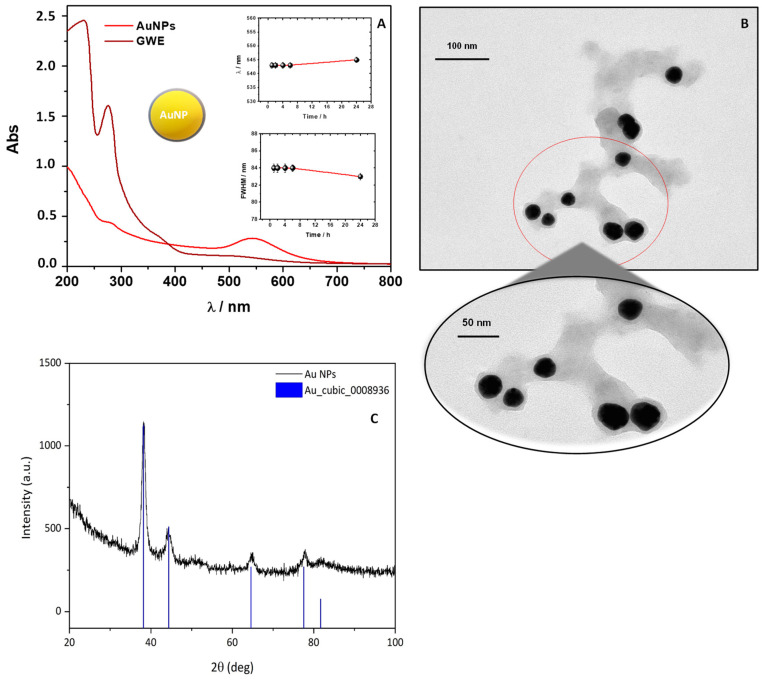
UV-Visible spectra of an AuNPs solution (dilution 1:5 from the stock solution), observing their typical SPR band (**A**). In the insets, the wavelength and FWHM time evolution registered in correspondence of the SPR are reported; TEM images of AuNPs at different magnification ratios (**B**); XRD diffraction patterns of AuNPs. Vertical bars: Bragg hkl reflections positions of the cubic Au crystal structure (ICSD code #044362) (**C**).

After mixing an appropriate amount of GWE with a HAuCl_4_ solution, the formation of AuNPs was observed. Furthermore, as noticed in our previous works focused on the green synthesis of AuNPs, the color of this solution changed from light yellow to violet, suggesting the AuNPs formation [12,13,14]. Indeed, the color of the gold-based solutions is due to the surface plasmon resonance (SPR) band that, in this case, was detected at 550 nm (Figure 2A). More specifically, to find the best experimental conditions, the AuNPs synthesis was followed by adopting different incubation times, from 1 h to 24 h.

Particularly, the insets of Figure 2A report the AuNPs SPR band time evolution focusing the attention on wavelength position, read at the maximum absorption values of the SPR band, along with the correspondent Full Width at Half Maximum (FWHM).

As described by Diegoli et al. [29], the position and shape of the SPR band are strongly related to particle size, dispersity, and degree of aggregation. The authors reported that the maximum of the SPR band moves towards longer wavelengths at increasing the particle size. While an increase in FWHM is related to the increase of particle dispersity [29]. Therefore, the absence of significant changes in these parameters over the contact time suggests that the AuNPs formation occurs rapidly without showing important aggregation phenomena.

Moreover, the symmetric shape of the SPR signal and the related FWHM reveal a low degree of polydispersity. On this ground, 1 h was adopted and proposed as the best contact time for the AuNPs’ redox synthesis attributable to the main components of GWE, mostly polyphenols, which reduce Au (III) to Au (0), as already reported in the literature [11,16]. Accordingly, by looking at the UV-Vis spectrum of the obtained colloidal AuNPs dispersion (Figure 2A), besides the clear SPR signal, a band around 280 nm is also visible and ascribable to the presence of phenols from GWE that acted as stabilizing agents, capping the surface of AuNPs [12]. This finding was unambiguously established by superimposing the GWE UV-Vis spectrum with the related AuNPs one, as reported in Figure 2A. The correspondence of this signal at 280 nm, present both in the GWE and AuNPs spectrum, confirmed the role of phenols as stabilizer agents in water [12].

### 3.2. Morphological and Chemical investigation of AuNPs

#### 3.2.1. TEM and XRD Analyses

TEM investigation revealed the formation of uniformly distributed polyhedral-shaped AuNPs (Figure 2B), with an average size of 30 ± 5 nm. Interestingly, TEM images evidenced the organic–inorganic hybrid nature of the synthesized AuNPs by showing a thin organic layer around them, ranging between 5 and 8 nm, which might provide stabilization and act as a nucleation site, as already reported by Nirmala et al. [11]. The qualitative phase analysis of the corresponding XRD pattern revealed the presence of the cubic Au phase (ICSD code #044362) (Figure 2C) [26].

#### 3.2.2. ATR-FTIR Spectroscopic Measurements

**Figure 3:**
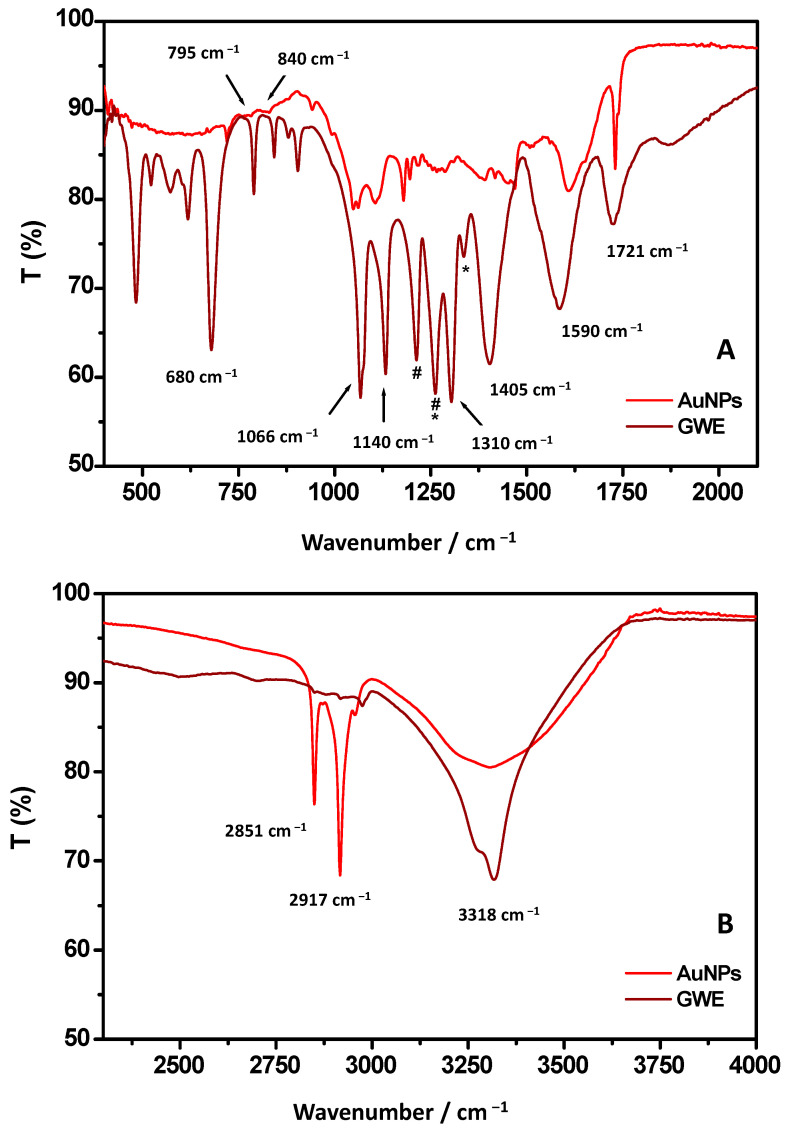
ATR-FTIR spectra of AuNPs and raw GWE referred to 500–2000 cm^−1^ (**A**) and 2500–4000 cm^−1^ (**B**) wavenumber regions. The signals at 1200 and 1252 cm^−1^ (indicated with * and #) are due to vibrations of C–O and C-OH groups, respectively, attributed to hydroxyflavonoids.

To better identify the chemical nature of biomolecules bound to the surface of the gold nanoparticles, ATR-FTIR measurements were performed (Figure 3A,B). As the first step, the FTIR analysis was focused on GWE. The related FTIR spectrum showed a sharp band centered at 3318 cm^−1^ assigned to stretching vibration of O-H from phenols groups.

The peaks around 2900 cm^−1^ correspond to C-H groups vibrations, as usually observed for phenols [12,30]. Accordingly, the CH_3_ out of plane bending and scissoring are observed at 1370 cm^−1^, and 1310 cm^−1^, respectively [31]. The band at 1721 cm^−1^, already observed by Gubitosa et al. [12], was identified as stretching vibration of C=O from aldehydes, ketones, carboxylic acids, and esters groups. The aromatic C-C stretching was observed at about 1590 cm^−1^, and it can be related, once again, to the presence of the phenolic compounds. Indeed, a signal around 1600 cm^−1^ is usually attributed to C=C–O deformation of the heterocyclic C-ring. A peak at 1143 cm^−1^, corresponding to aromatic C-H stretching, and the band at 795 cm^−1^ are also observed due to the CH_2_ rocking of phenolic compounds. In particular, the C–C–OH deformation of a phenol heterocyclic ring, attributed to the doublet at 840–795 cm^−1^, suggests the presence of tannins [32]. However, the contribution of amine moieties at 1590 cm^−1^ could also be taken into account, evidencing the occurrence, besides the main presence of phenols, of water-soluble amino acids and peptides [31]. The bands between 1405 cm^−1^ and 1066 cm^−1^ can be associated with C-H scissoring and bending of alkanes group and C-O stretching of alcohols, ethers, carboxylic acid, and ester groups (1150–1050 cm^−1^) [30]. Additionally, the signals at 1200 and 1252 cm^−1^ (indicated with #* and #) would be due to vibrations of C–O and C-OH groups, respectively, attributed to hydroxyflavonoids. The signal at 680 cm^−1^ was assigned to the C-H bending of the alkynes group [33]. Therefore, from the FTIR analysis of GWE, it was possible to assess the main presence of water-soluble phenols that might play a key role, as previously mentioned, in stabilizing AuNPs [11]. Indeed, the FTIR spectrum of the synthesized AuNPs showed the same signals previously observed for GWE, confirming the involvement of phenols in reducing Au (III) to Au(0) [32].

A deeper look at the AuNPs’ ATR-FTIR spectrum reveals that the band at 3318 cm^−1^ is broad, indicating the main involvement of the OH group, derived from phenols, in the AuNPs formation [33]. Oliveira et al. [33] suggested that free hydroxyl groups might interact with gold to stabilize AuNPs. In detail, the OH functional groups both in meta and para position and COOH of phenolic groups have been reported to act as stabilizing agents, capping the surface of nanogold [12,33]. At the same time, the band at 1590 cm^−1^ was shifted toward high wavenumbers, confirming the presence of C=C–O groups from phenols that, when on the surface of AuNPs, experimented a novel chemical environment due to the interaction of OH with AuNPs surface.

The band at 1721 cm^−1^, identified as stretching vibration of C=O from aldehydes, ketones, carboxylic acids, and esters groups, appeared sharp. Furthermore, the ratio between the relative intensities of bands at 1590 and 1721 cm^−1^ changed in favor of the latter [12,16], confirming the COOH group presence in the organic layer bound to AuNPs, although, probably, the C=O groups, as suggested by Ismail et al. [16], did not directly bind to the AuNPs surface. Indeed, Raota et al. [34] reported that OH moieties in phenols could form intramolecular H-bonds with C=O during the metal reduction step, forming a quinoidal species. Therefore, it is possible that, due to the main involvement of hydroxyl groups with Au surface, H-bond between OH and C=O did not occur, as suggested by the sharp IR C=O vibration. These results confirm that OH and O=C-OH functional groups might have interacted with the gold surface, rendering AuNPs highly stable [16]. Accordingly, important changes in band position and relative intensity were also observed in the wavenumber range 1200–1405 cm^−1^ due to the coordination of phenols. Furthermore, the signal at 680 cm^−1^ attributed to the C-H bending of the alkyne group moves to a higher wavenumber [33]. Interestingly, the relative intensities of C-H vibrations at about 2900 cm^−1^, at 2851 and 2917 cm^−1^ are more pronounced with respect to the same signals from GWE. This result could be attributed to a different arrangement of phenols when on the AuNPs’ surface [12,13].

#### 3.2.3. XPS Analysis

**Figure 4:**
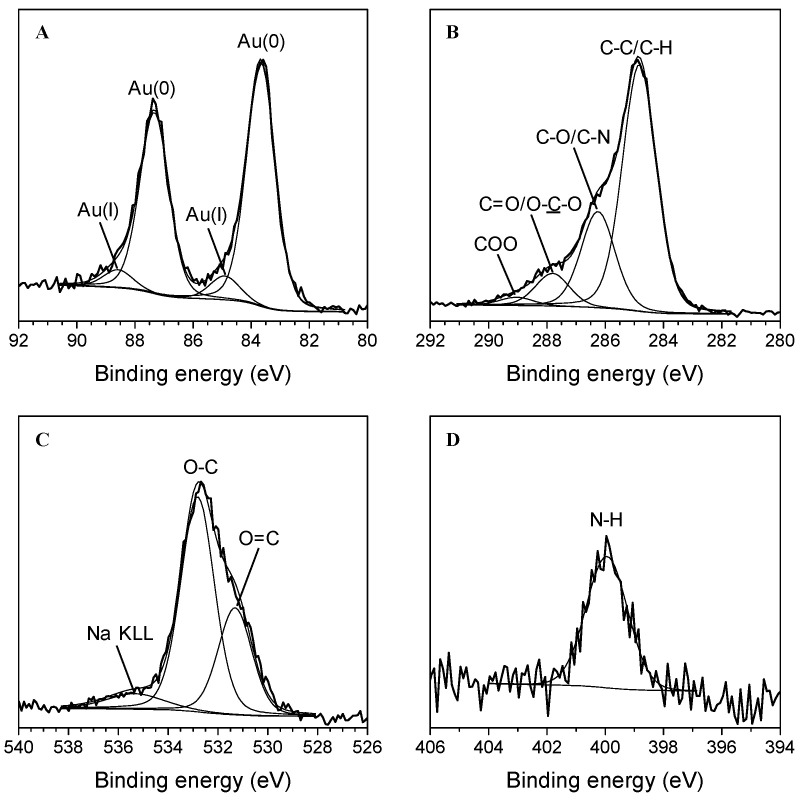
High-resolution XPS spectra of the AuNPs: Au 4f (**A**), C 1s (**B**), O 1s (**C**), and N 1s (**D**) spectra.

XPS characterization was carried out to better investigate the surface chemical composition of the Au nanoparticles. XPS analyses revealed that the surface atomic concentrations of carbon, oxygen, nitrogen, gold, sodium, and potassium are about 70, 25, 2, 0.5, 2, and 0.5%, respectively. Figure 4A displays the nanoparticles’ high-resolution Au 4f XPS spectrum, where the curve-fitting procedure has been applied to discriminate the peak components. Two doublets were found: the first is attributed to Au(0) (BE Au 4f_7/2_ = 83.6 ± 0.2 eV and BE Au 4f_5/2_ = 87.3 ± 0.2 eV, area percentage = 90%) and the second is ascribed to Au(I) (BE Au 4f_7/2_ = 84.9 ± 0.2 eV and BE Au 4f_5/2_ = 88.6 ± 0.2 eV, area percentage = 10%) [14,35,36]. The C 1s spectrum can be curve-fitted with four components ascribed to C-C/C-H (284.8 ± 0.2 eV, 65%), C-O (286.3 ± 0.2 eV, 25%), C=O/O-C-O (287.9 ± 0.2 eV, 8%) and COO (289.1 ± 0.2 eV, 2%) functional groups (Figure 4B) [12]. The O 1s signal presents two components at 531.3 ± 0.2 eV and 532.8 ± 0.2 eV due to O=C and O-C moieties, respectively (Figure 4C) [12]. Finally, the N 1s spectrum can be curve-fitted with only one component (399.9 ± 0.2 eV) assigned to amine groups (Figure 4D) [12]. As a whole, the XPS analysis clearly confirmed the reduction of Au (III) to Au (0) to form AuNPs surrounded by an organic shell that, according to the detected elements, corroborated the presence of phenols [12], denoting better the hybrid nature of AuNPs. The detection of amino moieties probably revealed the slight contribution of amino acids [12], as already observed during the ATR-FTIR analysis.

### 3.3. AuNPs Stability: Temperature, pH, and Salt Effects

#### 3.3.1. Thermostability

First, to assess the thermostability of the AuNPs after their synthesis, the experiments were performed by exposing a nanogold solution to a ramp of temperature from 10 to 90 °C (Figure S1), monitoring the wavelength position (λ) (Figure S1A) and the FWHM (Figure S1B) of the SPR at each temperature value. Indeed, as previously described and according to Diegoli et al. [29], the position and shape of the SPR band are strongly related to particle size, dispersity, and degree of aggregation. The obtained results revealed that the λ was the same from 10 to 80 °C and, at the same time, the correspondent FWHM slightly changed in this temperature range, suggesting the AuNPs’ thermostability. At 90 °C, a significant shift of λ and FWHM was observed, probably due to a slight AuNPs aggregation, indicative of a polydisperse sample [12,13,14].

#### 3.3.2. pH Effects

Different pH values, ranging from 2 to 12, were adopted to assess the influence of the pH on the AuNPs stability. For this purpose, NaOH and HCl were used to adjust the pH values of the AuNPs aqueous solution. Once again, by observing the λ and FWHM of the SPR of AuNPs, the results reported in Figure S2 were obtained. In this case, important changes were detected both in the λ position and FWHM. Slight changes were detected in the pH range from 4 to 12 by observing the slight decrease of both λ position and FWHM. So, certain stability of AuNPs can be supposed in this pH range. On the other hand, at pH < 6, the λ and FWHM moved towards higher values (Figure S2A,B), and a very important change was observed, especially at pH 2. Indeed, the SPR signal red-shifted from 543 nm (pH 6) to 547 nm (pH 2), denoting the contribution of agglomerated AuNPs [12,13,14]. Accordingly, at the same time, the related FWHM increased from 85 to 90 nm, indicating an increased AuNPs dispersity. Indeed, Gubitosa et al. [12,13,14] suggested that the excess of H^+^ under acid conditions could affect the AuNPs’ surface and charges, so AuNPs tended to aggregate more easily, obtaining a larger final particle size. The zeta potential and size measurements were performed to better clarify these results, (Figure S2C). Diegoli et al. [29] reported that the zeta potential of a colloidal solution is indicative of its stability. More specifically, the greater the zeta potential, the higher the repulsion between the particles. An empirical rule reports that colloidal suspensions showing zeta potentials in the range −30 mV and +30 mV are generally unstable while are considered stable when the zeta potentials are more positive/negative than ±30 mV [29]. In the water medium at pH 6, the studied AuNPs present a negative charge (−40 mV), with a zeta potential value indicating their good stability. On the other hand, the zeta potential values collapse to almost zero under pH values lower and greater than 6. Despite these findings, the size measurements did not reveal aggregated AuNPs at pH > 6, showing a hydrodynamic diameter of 120 ± 30 nm. The results agreed with those obtained during the study of λ position and FWHM. The presence in the solution of ions derived from NaOH and HCl, which, balancing the layer of charges around AuNPs, lower their zeta potential, can explain these findings [37]. So, this change in nanoparticle surface charge was likely due to the strong effect of ions in the solution that perturbed the zeta potential. Only at pH 2, as previously anticipated, the size increases to 1500 nm, suggesting the presence of AuNPs clusters as expected by the measured zeta potential (0 mV). Consequently, the electrostatic clustering due to the annihilation of zeta potential can determine the AuNPs’ increased size [37]. On the other hand, it is possible to assess that the presence of functional groups coming from the main components of GWE, such as the carboxylic moieties of phenols, and present on the AuNP surface, conferred a negative electrical charge in water at pH 6. On the other hand, at pH values below 6, the organic moieties were in their protonated form, and the AuNPs rapidly tended to aggregate.

#### 3.3.3. Salt Effects: The Case of NaCl

Colloidal gold was mixed with a highly concentrated NaCl solution, so Na^+^ and Cl^−^ ions interacted with the AuNPs’ surface. As previously reported, it has been well demonstrated that both the λ and FWHM of the SPR signal were diagnostic to infer information about the state of aggregation and dispersity of AuNPs. Figure S3 shows the NaCl effect on these two parameters. By increasing the salt concentration from 0.01 to 3 M, a clear red-shift of the SPR band was observed (Figure S3A), together with an increased dispersity (Figure S3B) indicative of less stable AuNPs [12,13,14]. Increasing the salt concentrations reduces the electrostatic double layer (EDL) repulsive energy between particles [36]. The phenomenon was better studied by changing both the cations’ and anions’ nature (Figure S5). Figure S4A,B show the effect of cations (by fixing Cl^−^ as an anion), having different sizes and charges, on λ and FWHM. For this purpose, three model salt concentrations were adopted: 0.01, 0.5, and 3 M. Clearly, by increasing the monovalent cation size from Li^+^ to K^+^, both the wavelength and FWHM increased, suggesting that the dispersity and aggregation of AuNPs were affected. The effect was more pronounced at a higher salt amounts. The different behavior of Li^+^, Na^+^, and K^+^ could be explained in terms of hydration. Specifically, their hydration radii are K^+^ =2.32 Å, Na^+^ =2.76 Å, and Li^+^ = 3.4 Å [20]. Li^+^ strongly holds water molecules due to its smaller size with respect to K^+^ or Na^+^, preventing the association with the AuNPs surface and affecting the EDL thickness slightly. Indeed, the effect becomes evident only by increasing the Li^+^ concentration [38]. Accordingly, by adopting bivalent cations, such as Mg^2+^ and Ca^2+^, the effect was more evident, and, particularly, by increasing the size of cations from Mg^2+^ to Ca^2+^, the aggregation was greater. This behavior could be explained by considering that bivalent ions come closer to the surface of nanoparticles due to the presence of a stronger attraction. As a result, the thickness of the EDL decreased, and the nanoparticles came closer to each other, favoring the aggregation [38]. The different behavior observed in the presence of Ca^2+^ and Mg^2+^ could be explained once again in terms of the different hydration degrees of those ions. Mg^2+^, as observed in the case of Li^+^, tightly holds water molecules due to its smaller size with respect to Ca^2+^. Therefore, water molecules are less likely to dissociate from Mg^2+^ than Ca^2+^, hindering the binding with the negatively charged AuNPs [39]. Figure S4C,D report the same investigation changing the nature of anion, and, thus, the size, by adopting Cl^−^, Br^−^ and ClO_4_^−^. In this case, Na^+^ was fixed as the cation. The absence of significant changes was detected. The finding can be easily rationalizable by considering the negative charge of AuNPs in an aqueous medium that should be more affected by the presence of cations than anions, disturbing the surface charges, thus favoring the aggregation of AuNPs.

### 3.4. AuNPs’ Photostability and Evaluation of the Theoretical SPF

**Figure 5:**
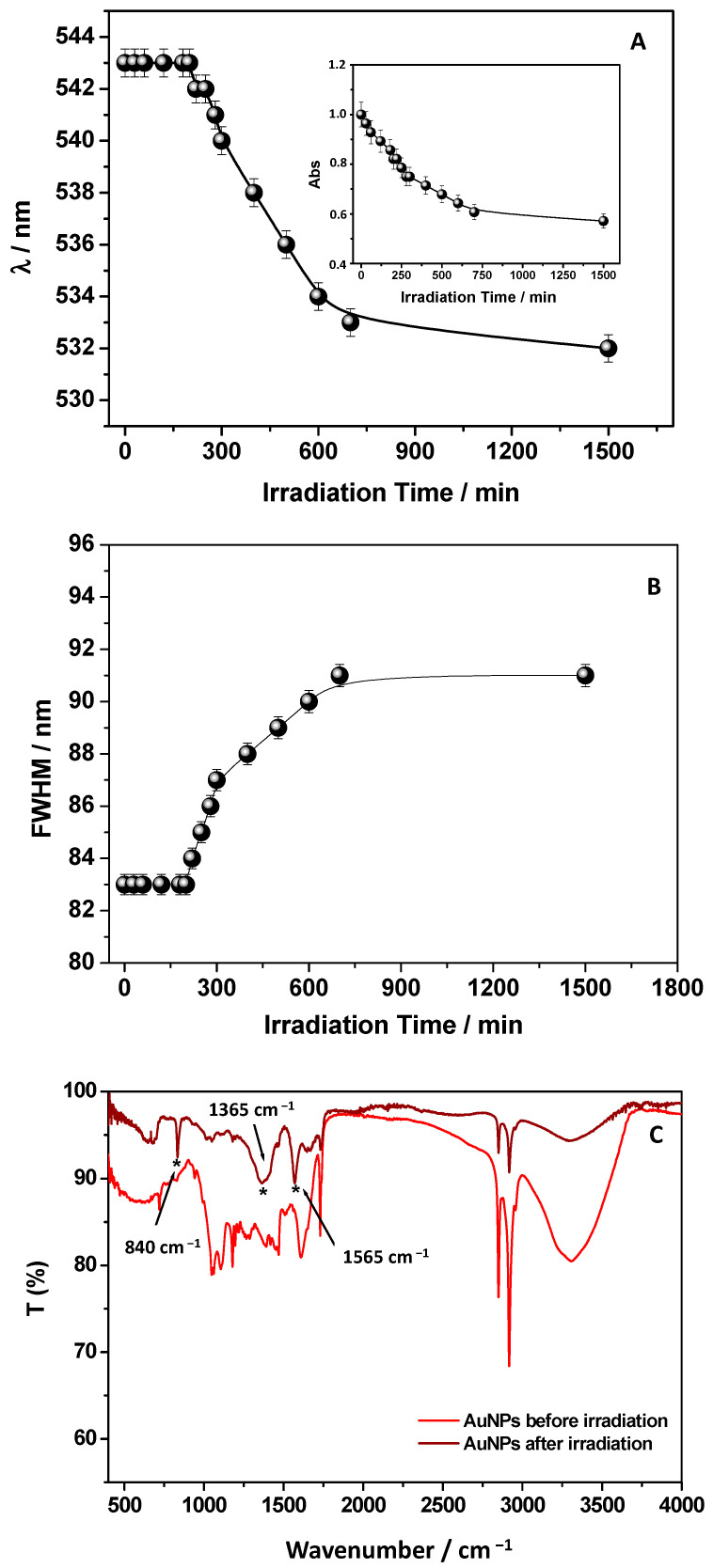
Effect of irradiation time, by using a sun simulator lamp, on wavelength position (**A**) and FWHM (**B**) of AuNPs SPR. The inset (**A**) shows the intensity of the SPR band in terms of absorbance values read at the maximum of SPR at several irradiation times. ATR-FTIR spectra of AuNPs before and at the end of irradiation. Bands indicated with * at 840, 1365, and 1565 cm^−1^ suggest the formation of surface carbonates, and the finding agrees with the well-known mechanisms of phenol photodegradation (**C**).

The AuNPs’ ability to screen the solar radiation due to the organic layer present on their surface was investigated. As the first step, the nanoparticle photostability was assessed, and a sun simulator lamp was used to monitor the SPR signal. On the one hand, the plasmonic band resulted stable until 200 min; on the other hand, by elapsing the irradiation time over 200 min, the lack of stability was observed. Indeed, the wavelength position (Figure 5A) moved toward lower values indicating the disassembling of AuNPs that reduced their size. At the same time, the FWHM values (Figure 5B) increased due to the increased dispersity of AuNPs. The absorbance intensity of the SPR (inset in Figure 4A) occurred significantly reduced, confirming the AuNPs degradation.

Considering that a natural extract mediated the AuNPs’ formation mechanism, the gold nanoparticles were synthesized by assembling Au(0) nuclei obtained through reduction from Au(III) by phenols. Therefore, the phenol’s degradation on the gold surface that acted as stabilizing agents could be responsible for the AuNPs disassembling, preventing their collapse in water. Collecting the ATR-FTIR spectrum of AuNPs at the end of the irradiation time further confirmed the finding (Figure 5C). It can be clearly observed that comparing the FTIR spectrum of AuNPs after their irradiation with the spectrum registered before the sun lamp exposure shows important band intensity and position changes. Due to the organic layer degradation, the relative intensities in the spectrum appeared to have changed. In particular, the bands in the region 1000–1600 cm^−1^ occurred greatly reduced in intensity, and new bands indicated with asterisks at 840, 1365, and 1565 cm^−1^ emerged. Mino et al. [40] attributed the bands at around 1560 and 1380–1360 cm^−1^ to the formation of surface carbonates, and the finding agrees with the well-known mechanisms of phenol photodegradation: a phenol is first converted to catechol, p-benzoquinone, and hydroquinone, and then to short-chain acids with the final formation of CO_2_ and water [40]. Not surprisingly, the formation of bubbles in the irradiated solution was observed during the experiment. The change of the C–C–OH deformation of the phenols heterocyclic ring, which has been attributed previously at about 840 cm^−1^, confirmed the changes in the phenols structure. These results suggest that AuNPs could prevent sunlight damage by acting as a potential chemical filter. The presence of an organic phenols-based layer able to absorb sunlight could be responsible for this behavior. Not surprisingly, the UV-Vis spectrum of AuNPs, reported in Figure 2A, shows high absorption in the region below 400 nm indicating the AuNPs’ potential use as a sunscreen, preventing sunburn and other skin damage. On this ground, by adopting the procedure described by Gubitosa et al. [12], a simple method based on the use of absorption spectroscopy was applied in this work to infer the theoretical Sun Protection Factor (SPF) value. More specifically, to calculate the SPF in the range 290 nm−320 nm, Equation (1) was used. For example, by adopting the spectrum reported in Figure 2A corresponding to a dilution 1:5 obtained from the stock AuNPs solution 1 × 10^−11^ M, an SPF value of 5 was calculated, which could be potentially increased by increasing the AuNPs concentration. In this context, the proposed AuNPs could be considered as SPF boosters to increase, for example, the sunscreen properties of commercial products in preventing skin photodamage.

Interestingly, Xian et al. [41] reported that skin photodamage favors the Reactive Oxygen Species (ROS) UV-induced production, with the inactivation of NF-E2-related factor 2 (Nrf2). This factor, Nrf2, regulates the basal and induced expression of an array of antioxidant genes regulating oxidants’ physiological and pathophysiological outcomes [42]. In detail, the excessive ROS production by a high dose of UV light affects the Nrf2/antioxidant response pathway, reducing the antioxidant defense system and, thus, inducing cutaneous disorders [42]. Therefore, skin disorders could be prevented if sunscreen also acts as an antioxidant [12,13,14]. Simultaneously, if the same sunscreen acts against the tyrosinase enzyme, inhibiting melanin formation, a multifunctional nanosystem that also prevents hyper-pigmentation can be presented. Consequently, the antioxidant and tyrosinase inhibitor properties of the proposed AuNPs were also investigated during this work.

### 3.5. Antioxidant Properties of AuNPs

**Figure 6:**
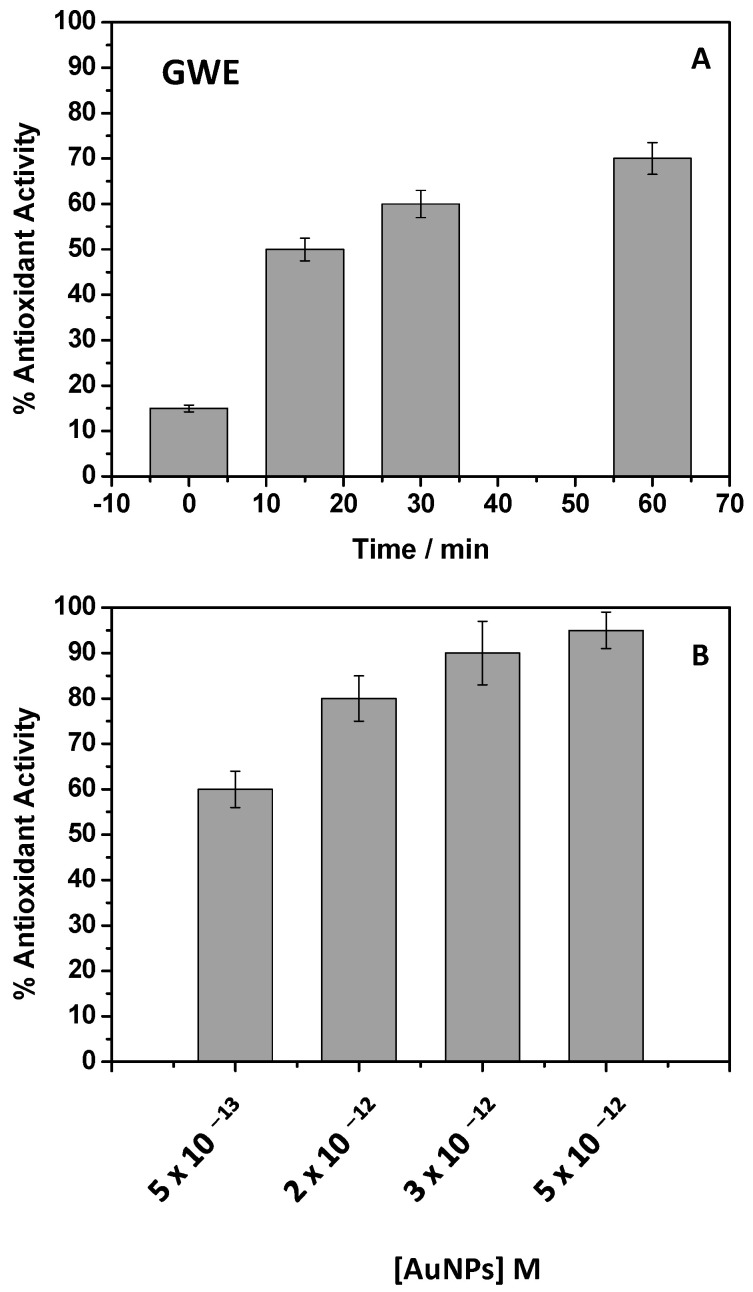
Percent of the antioxidant activities of raw GWE (2 mg/L), calculated at different incubation times in the presence of ABTS (**A**) and AuNPs at different concentrations (**B**).

Phenols are considered the main natural compounds that induced the AuNPs formation, acting as capping and stabilizing agents in water medium. Indeed, if these molecules reduce Au(III) solutions, they work as reductant agents and thus as antioxidants. Accordingly, phenols are the most common antioxidant agents, and their properties are well known. So, the antioxidant action of AuNPs should be expected considering their hybrid inorganic/organic nature. For this purpose, the ABTS assay was performed to assess the AuNPs’ antioxidant ability. However, for the sake of comparison, the GWE antioxidant properties were also evaluated. The bleaching of the ABTS^•+^ in the visible spectrum region from 500 nm to 900 nm was thus monitored at different GWE dilution and time intervals. The degree of decolorization was determined by using Equation (2). According to previous studies investigating grape pomace’s properties [43,44,45], the results obtained as % of antioxidant activity from GWE are reported in Figure 6A.

In particular, different GWE %, from 100% to 0.002%, after dilution in water, were investigated. All the explored conditions showed high antioxidant activity, and the ABTS bleaching was observed after a few seconds. Only the lowest dilution, when the GWE was at 0.002%, and corresponding to a concentration of 2 mg/L, enabled us to follow the time color evolution (Figure 6A). After 30 min of contact time, the response appeared almost complete and similar to the result obtained after 1 h, showing a % of antioxidant activity of about 70%. After these considerations, the antioxidant behavior of AuNPs adopted at different dilutions was investigated (Figure 6B). In this case, a typical dose-response histogram was obtained by adopting an incubation time of 30 min, denoting that the ABTS^•+^ bleaching slowed down by diluting the sample. It should be underlined that despite the used AuNPs concentrations being very low, a high % of antioxidant activity, around 100%, was observed, confirming that the phenols on the AuNPs surface retain their antioxidant properties [12,13].

To better understand these results, further experiments were also performed in the presence of AuNPs and H_2_O_2_, like a model oxidant agent. Figure S5 shows the obtained results. Once again, the λ and FWHM values relative to the plasmonic signal were considered diagnostic and monitored (Figure S5A,B). Albeit the presence of a strong oxidant agent, as H_2_O_2_, used at 0.1 M, the λ and FWHM resulted constant for 90 min, indicating the AuNPs’ SPR band stability. However, to infer more information, the ATR-FTIR spectrum of AuNPs at the end of the experiments was also collected, and it is reported in Figure S5C.

Interestingly, as previously observed, the intensity of the FTIR spectrum was, as a whole, reduced, indicating partial phenols degradation. In fact, bands similar to those reported in Figure 5C, at 1560 and 1380–1360 cm^−1^, were detected, attributable to the oxidation of the phenolic molecules surrounding AuNPs, as before observed during the nanoparticles’ irradiation. The obtained results clearly indicated that the phenols around AuNPs worked by scavenging the hydrogen peroxide paying with their oxidation.

### 3.6. Tyrosinase Assay

**Figure 7:**
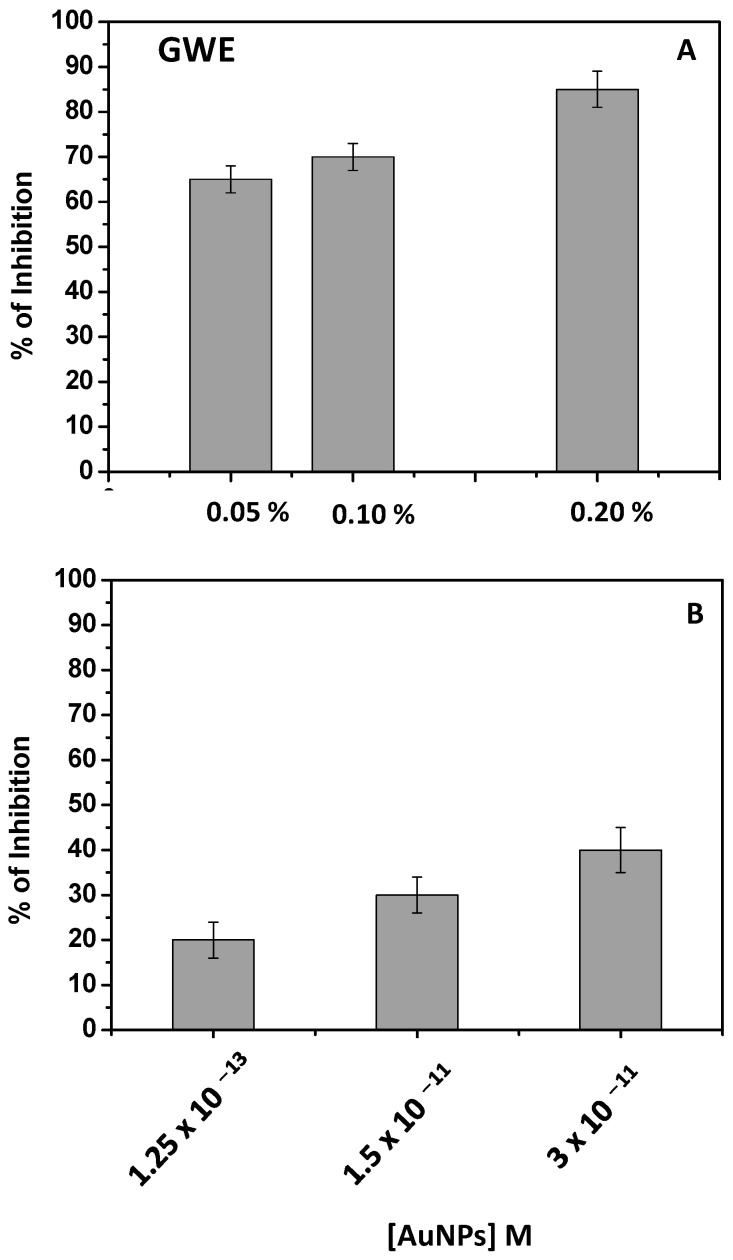
Percent of tyrosinase inhibition of raw GWE (2 mg/L) (**A**) and AuNPs (**B**) at different amounts.

Phenols can also act as tyrosinase inhibitors due to their nature as primary and secondary antioxidant agents. In particular, tyrosinase inhibition can occur through specific tyrosinase inactivators, called “suicide inactivators” (or mechanism-based inhibitors), or by means of “true inhibitors”, compounds belonging to four types: competitive, uncompetitive, mixed type (competitive/uncompetitive), and non-competitive [46,47]. Among this latter class of natural molecules, as secondary antioxidants, phenols, being able to chelate the copper, can competitively inhibit tyrosinase activity by indirectly hindering the formation of oxidant species [46,47]. Particularly, the tyrosinase inhibition is due to the chelation, by phenols, of copper present on the enzyme active site. Depigmentation is usually ascribed to the loss of enzyme activity [12,13,14]. Once again, the GWE activity was investigated as the first step, and the obtained results were reported in Figure 7A.

As evidenced by the antioxidant activity, the effect was already pronounced by adopting a very low % of GWE in water (2 mg/L). The incubation time was fixed at 30 min, and an important inhibition of the enzyme was observed. For example, by adopting GWE at 0.20%, the tyrosinase inhibition was quite complete.

Similar considerations, obtaining a dose-response histogram, should be applied when AuNPs were in use (Figure 7B), confirming, once again, that the tyrosinase inhibitor activity of phenols was retained on AuNPs. In this case, the effect of phenols was probably less pronounced with respect to GWE due to the lowest amount of phenols present on the AuNPs’ surface. It is likely that the phenols capping AuNPs act as chelating agents, partially hindering the chelation of copper by tyrosinase.

### 3.7. AuNPs Reduce H_2_O_2_-Induced Cytotoxicity in NHDFs

**Figure 8:**
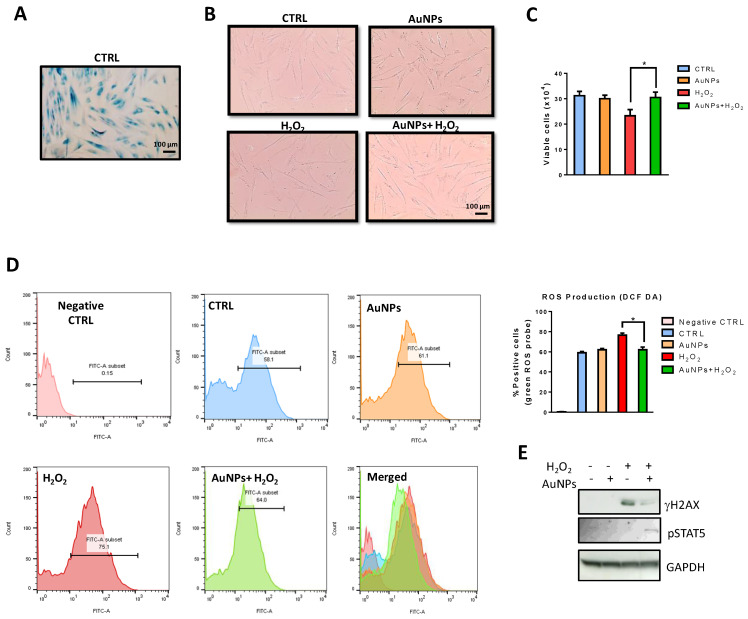
Effects of AuNPs in H_2_O_2_-exposed NHDFs. (**A**) Representative phase-contrast photomicrographs of SA-β-Gal positive cells. (**B**) Fibroblast morphology before and after treatments. Images are representative of cell cultures photographed using a phase-contrast microscope (100× magnification). (**C**) Histograms reporting viable cells (trypan blue negative) (**D**) Intracellular ROS scavenging activity of AuNPs in H_2_O_2_-exposed NHDFs. Histograms represent the percentage of cells positive for the DCF-DA probe. (**E**) Effects of AuNPs on H2AX and STAT5 phosphorylation were evaluated by western blot analysis. Data are presented as the mean ± standard deviation from triplicate experiments. * *p* < 0.05, compared with H_2_O_2_-treated cells.

Beta-galactosidase (beta-gal) at pH 6.0 has been widely used as a marker of cellular senescence in vivo. In vitro, it increases during the replicative senescence of fibroblast cultures (Figure 8A). Indeed, as reported in Figure 8B, the old fibroblast showed a large flattened morphology, also evident in nuclear shape. To determine whether AuNPs affect the viability of pre-senescent NHDFs exposed to H_2_O_2_-induced cellular stress, NHDFs were sequentially treated with AuNPs and H_2_O_2_, as described in the material and methods section, and cytotoxicity was evaluated using a Trypan Blue assay. As presented in Figure 8C, the pretreatment with AuNPs for 15 h significantly inhibited H_2_O_2_-induced cytotoxicity.

### 3.8. AuNPs Inhibit H_2_O_2_-Induced Oxidative Stress in NHDFs

Massive production of ROS or the inability to remove them can result in oxidative stress, whereby ROS promote the damage of proteins, lipids, and DNA leading to cellular stress or apoptosis and cellular senescence [48]. The present study investigated whether the protective effects of AuNPs against the well-known H_2_O_2_-induced cellular damage were associated with oxidative properties. For evaluating the ROS scavenging activity of AuNPs in H_2_O_2_, DCF-DA fluorescence intensity was assessed. DCF-DA fluorescence intensity, which is an indicator of intracellular ROS levels, was studied in AuNPs-pretreated NHDFs with or without H_2_O_2_ exposure. DCF-DA intensity increased in H_2_O_2_-exposed cells compared with the non-exposed control group (Figure 8D). However, this effect was reduced in NHDFs pretreated with AuNPs.

### 3.9. Reduction of H_2_O_2_-Mediated DNA Damage by AuNPs

As oxidative stress-induced damage to DNA results in lesions that are responsible for the loss of cell viability [49], H_2_O_2_-mediated damage to NHDFs cell DNA was detected using western blot analysis. The levels of phosphorylation of nuclear histone H2AX (Ser139) (p-γH2A.X), a sensitive marker for DNA double-strand break (DSB) formation, increased in the H_2_O_2_-treated cells, as shown by western blotting (Figure 8E). However, pretreatment with AuNPs resulted in a significant decrease in the expression of γH2AX. To gain insight into molecular mechanisms by which AuNPs mediated H_2_O_2_-induced DNA damage, we evaluated the phosphorylation levels of STAT5, important for activation of the ATM DNA damage pathway. Indeed, it has been reported that pSTAT5 dependent upregulation of RAD51, recruiting it to repair DSBs by homologous recombination, promoting anti-apoptotic activity of BCL-XL, and delaying cell cycle progression [50]. We observed a significant increase in STAT5 phosphorylation by western blot analysis when NHDFs were sequentially treated with AuNPs and H_2_O_2_ (Figure 8E).

Overall, the results suggest that AuNPs have a protective property against DNA damage induced by H_2_O_2_ treatment.

### 3.10. GP as Adsorbent Material to Remove EPs: A Preliminary Study

As proof of concept, CIP and TC were chosen as examples of emergent contaminants for testing the ability of exhausted grape pomace to be used as an adsorbent for cleaning polluted water. For this purpose, the UV-Visible spectra of CIP and TC were considered diagnostic for monitoring their adsorption [18,25], thus following their removal from water. Using 30 mg of GP, the % of adsorption of these pollutants changed from 20% in the first 15 min of contact time to 50% after 60 min. For example, Scheme 1 reported the case of CIP, and by observing the CIP UV-Vis absorption spectrum, it is possible to see the absorbance intensity decrease at increasing the contact time. The samples were further left for 24 h to reach their total removal. A faster purification was obtained by incrementing the adsorbent amount at 100 mg by maintaining constant the CIP concentration (5 mg/L), leaving clean water by removing 100% of this EP. These findings suggest the presence of free active sites onto the exhausted GP adsorbent surface able to host the pollutant [18]. However, further and deeper investigations are needed to characterize the whole adsorption process with the aim to better evidence this novel possible use of GP.

## 4. Conclusions

Under Green Chemistry and Circular Economy principles, advances in the synthesis of AuNPs, starting from a water-based polyphenolic extract derived from grape pomace (GWE), a typical agricultural waste, were presented during this work. After obtaining in one-pot reaction the AuNPs, a careful characterization by using several complementary techniques was accomplished. A comprehensive investigation was performed by evaluating the thermal stability and photostability and exploring the role of pH and ionic strength on AuNPs’ stability to show their physical and chemical properties. The SPR signal was constantly monitored for the purpose. The finding was confirmed by dynamic light scattering and Zeta Potential measurements. For proposing the AuNPs in nanomedicine and cosmetic fields, the antioxidant and tyrosinase inhibition features of AuNPs were successfully evaluated by using ABTS and an enzymatic test, respectively. As stated from preliminary in-vitro experiments, AuNPs attenuated H_2_O_2_-induced growth inhibition and exhibited scavenging activity against the intracellular ROS. AuNPs also inhibited phospho histone γH2A.X expression and induced the STA5 phosphorylation, suggesting that they can prevent H_2_O_2_-induced cellular DNA damage and apoptotic cell death. Altogether, these data suggested that AuNPs may be considered a potential ingredient in anti-aging skin products. Preliminary results about the reuse of exhausted grape wastes after the extraction of GWE were also proposed for a more sustainable cyclic use of wastes. For this purpose, the removal of emerging pollutants such as Ciprofloxacin and Tetracycline was performed from water, opening a novel horizon in this field, and presenting the dual life and use of grape pomace.

## Data Availability

All of the data is contained within the article and the supplementary materials.

## Supplementary Materials

The following supporting information can be downloaded at: https://www.mdpi.com/article/10.3390/antiox11050994/s1, Figure S1: Effect of temperature on wavelength position and FWHM of AuNPs SPR; Figure S2: Wavelength position and FWHM of the AuNPs SPR band, and Zeta Potential at several pH values; Figure S3: Effect of salt concentration (NaCl) on wavelength position and FWHM of AuNPs SPR band; Figure S4: Effect of different salts on wavelength position and FWHM of AuNPs SPR band; Figure S5: Effect of H_2_O_2_ on wavelength position and FWHM of AuNPs SPR band, and FTIR-ATR spectra of AuNPs before and in presence of H_2_O_2_.
