# Supplementary material for: The “End Life” of the Grape Pomace Waste Become the New Beginning: The Development of a Virtuous Cycle for the Green Synthesis of Gold Nanoparticles and Removal of Emerging Contaminants from Water
Source: Antioxidants (Basel). 2022 May 19;11(5):994. doi: 10.3390/antiox11050994 (PMC9137750; doi:10.3390/antiox11050994)
Supplement: Supplementary file 1 [file antioxidants-11-00994-s001.zip › antioxidants-1713570-supplementary.pdf]

ELECTRONIC SUPPORTING INFORMATION

The "end life" of the grape pomace waste become the new beginning: the development of a virtuous cycle for the green synthesis of gold nanoparticles and removal of emerging contaminants from water

Jennifer Gubitosa[a], Vito Rizzi[a], Anna Laurenzana[b], Francesca Scavone[b], Elena Frediani[b], Gabriella Fibbi[b], Fiorenza Fanelli[c], Teresa Sibillano[d], Cinzia Giannini[d], Paola Fini[e], Pinalysa Cosma[a*]

[a]Università degli Studi "Aldo Moro" di Bari, Dipartimento di Chimica, Via Orabona, 4- 70126 Bari, Italy;

[b]Dipartimento di Scienze Biomediche Sperimentali e Cliniche "Mario Serio" Viale Morgagni 50 - 50134, Florence, Italy;

[c]Consiglio Nazionale delle Ricerche, Istituto di Nanotecnologia (CNR-NANOTEC) c/o Dipartimento di Chimica, Università degli Studi "Aldo Moro", Via Orabona, 4 - 70126 Bari, Italy;

[d]Consiglio Nazionale delle Ricerche CNR-IC, UOS Bari, Via Amendola,122/O 70126 Bari, Italy

[e]Consiglio Nazionale delle Ricerche CNR-IPCF, UOS Bari, Via Orabona, 4- 70126 Bari, Italy;

*Corresponding Authors: Prof. Pinalysa Cosma
Università degli studi di Bari "Aldo Moro"
Dipartimento di Chimica, Via Orabona 4-70126 Bari, Italy
Tel: +390805443443

E-mail pinalysa.cosma@uniba.it

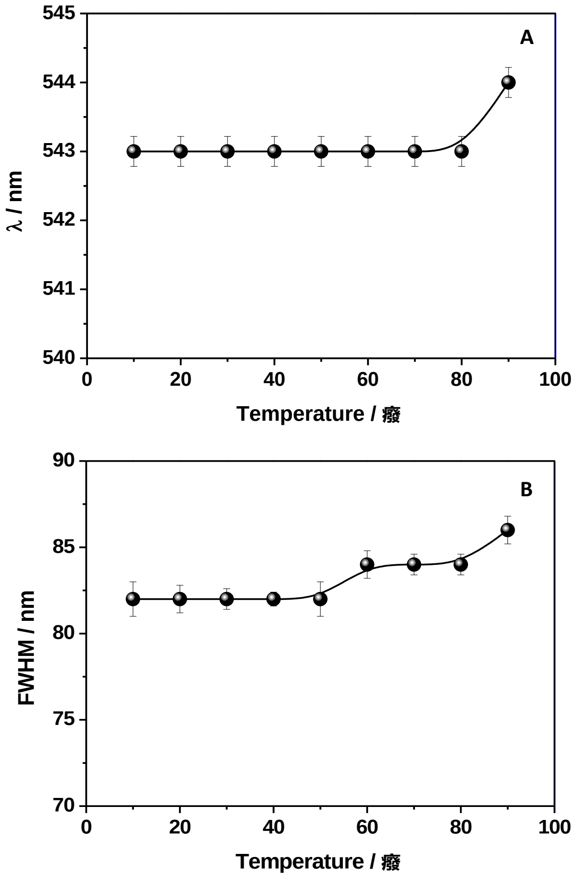

Figure S1: Effect of several temperature values (10-90°C) on wavelength position (**A**) and FWHM (**B**) of AuNPs SPR band.

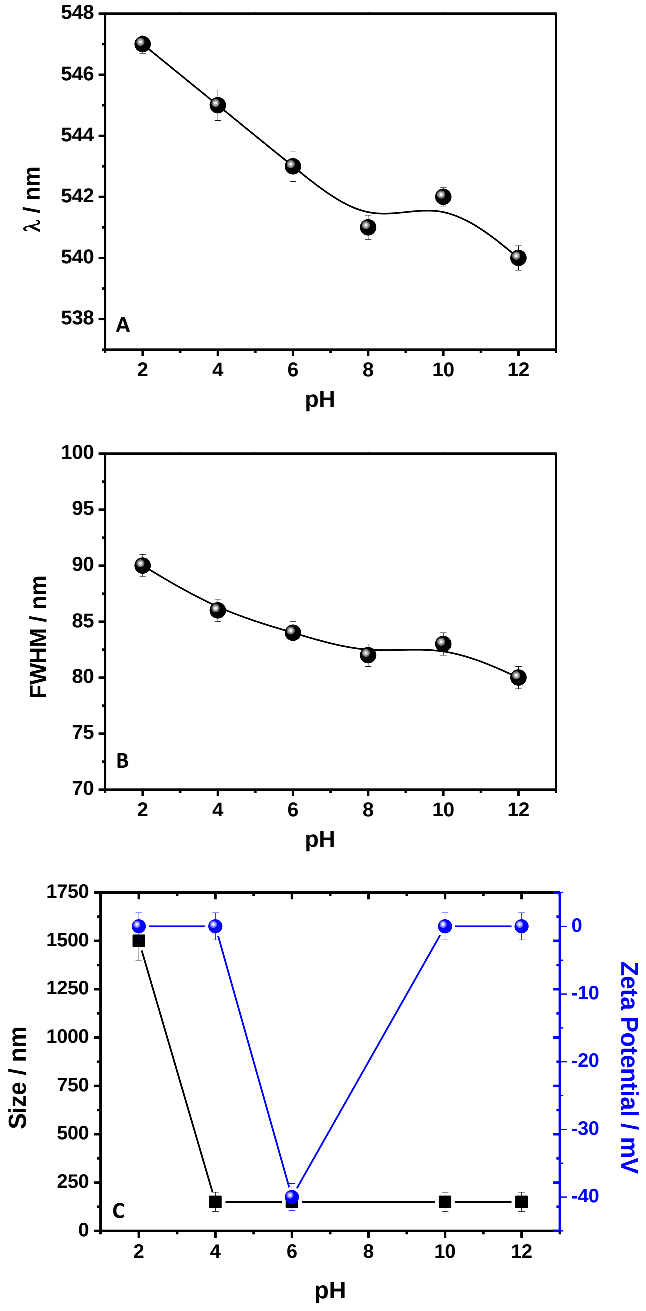

Figure S2: Wavelength position (**A**) and FWHM (**B**) of the AuNPs SPR band at several pH values (2-12); AuNPs size and Zeta Potential at several pH values (**C**).

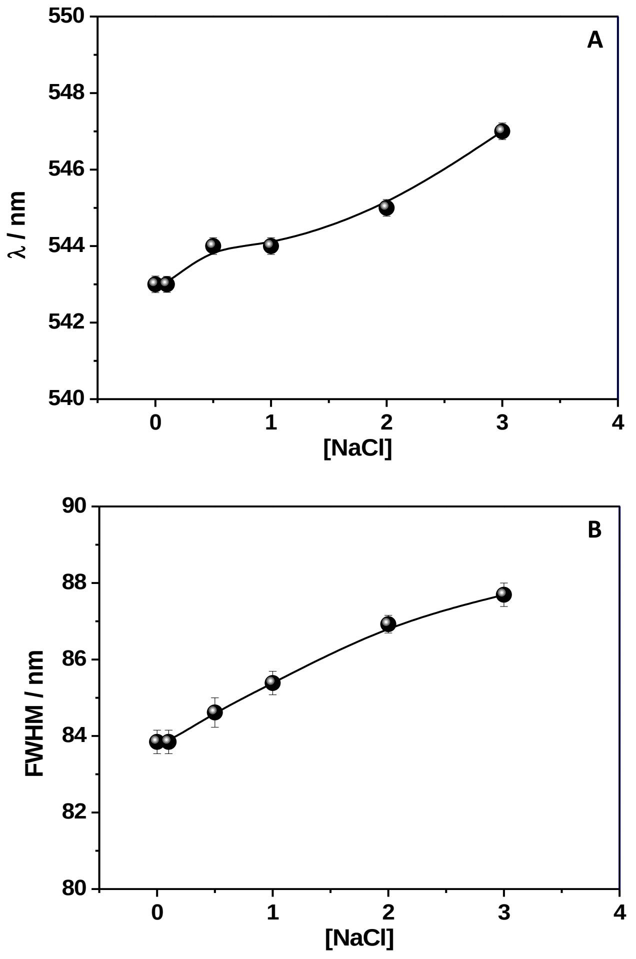

Figure S3: Effect of salt concentration (NaCl) on wavelength position (**A**) and FWHM (**B**) of AuNPs SPR band.

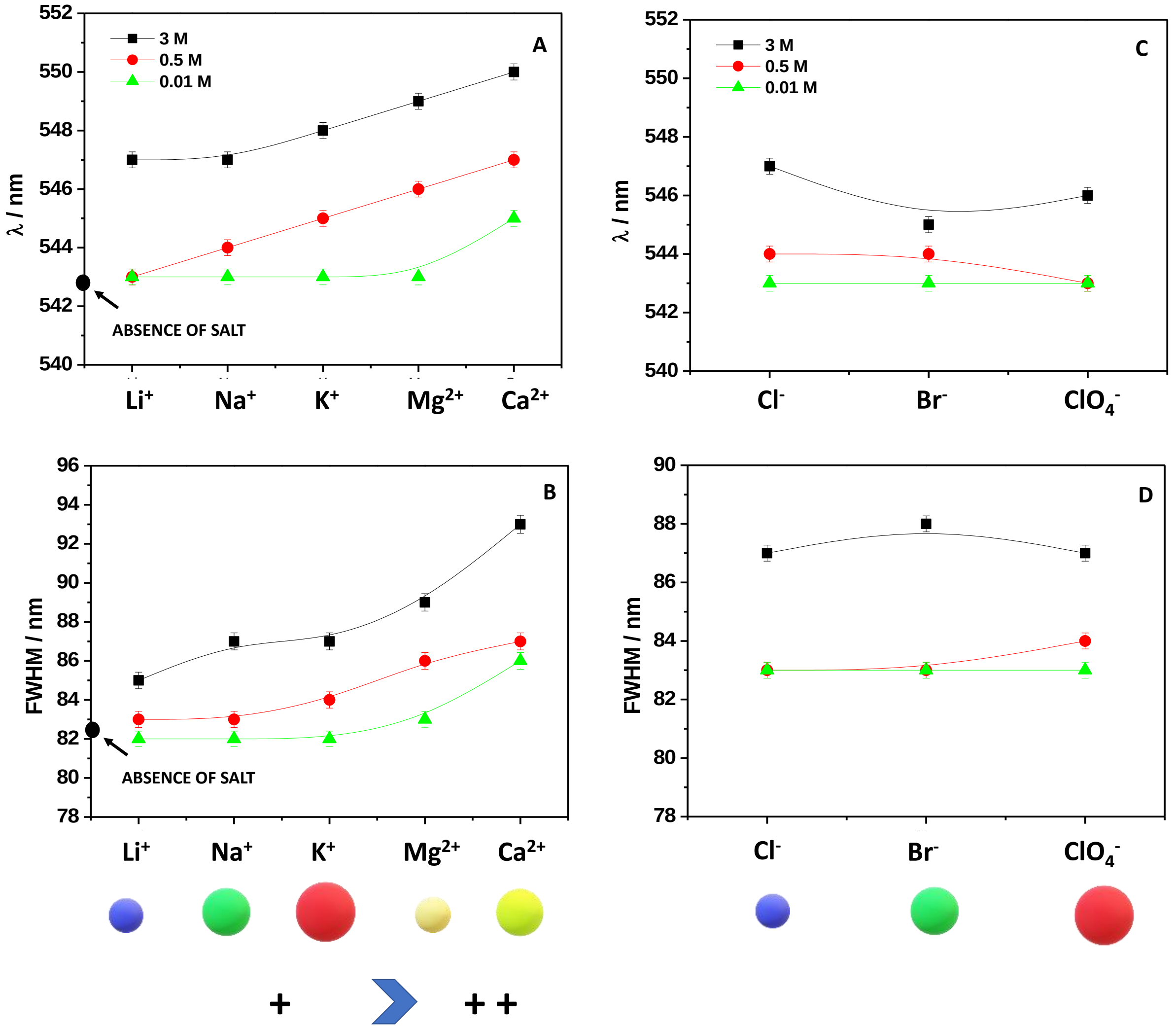

Figure S4: Effect of different salts on wavelength position (**A**, **C**) and FWHM (**B**, **D**) of AuNPs SPR band, evaluating the role of cations (**A**, **B**) and anions (**C**, **D**).

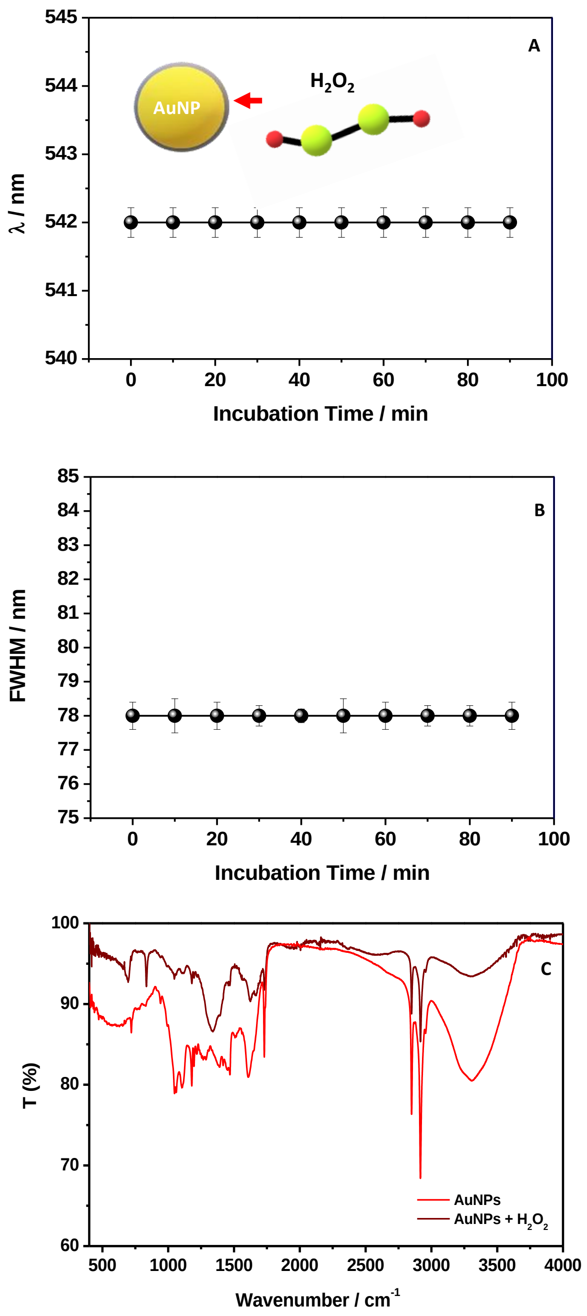

Figure S5: Effect of H_2O_2 (0.1 M) at different incubation time on wavelength position (**A**) and FWHM (**B**) of AuNPs SPR band; FTIR-ATR spectra of AuNPs before and at the H_2O_2 mediated oxidation.
